# Preparation and Characterization of Fibrous Alumina and Zirconia Toughened Alumina Ceramics with Gradient Porosity

**DOI:** 10.3390/nano12234165

**Published:** 2022-11-24

**Authors:** Eszter Bódis, Kolos Molnár, János Móczó, Zoltán Károly

**Affiliations:** 1 Institute of Materials and Environmental Chemistry, Research Centre for Natural Sciences, H-1117 Budapest, Hungary; 2 Department of Polymer Engineering, Faculty of Mechanical Engineering Budapest, University of Technology and Economics, H-1111 Budapest, Hungary; 3 ELKH-BME Research Group for Composite Science and Technology, H-1111 Budapest, Hungary

**Keywords:** ZTA ceramic, fibre, SPS, gradient porosity

## Abstract

This paper investigated a synthesis process for highly porous Al_2_O_3_, Y-ZTA, and Ce-ZTA ceramic nanocomposites with gradient microstructure and improved mechanical properties. Ceramic nanofibres were synthesized as the starting material. The gradient microstructure was developed during spark plasma sintering using an asymmetric graphite arrangement that generated significant temperature differences (80–100 °C) between the opposite sides of the samples. Structural and mechanical properties of the fibrous ceramic composites were investigated. The effect of the temperature gradient on properties was also discussed. While the asymmetric configuration resulted in a gradient porosity, reference samples fabricated in standard graphite configuration were uniformly porous. The gradient structure and the ZrO_2_ addition led to improved hardness and compression strength of the sintered samples. However, the opposite sides of the samples exhibited considerable variations in both microstructure and in terms of properties. The upper part of the Ce-ZTA ceramic showed a highly porous structure with 18.2 GPa hardness, while the opposite side was highly densified with 23.0 GPa hardness. Compressive strength was 46.1 MPa and 52.1 MPa for Y-ZTA and Ce-ZTA sintered at 1300 °C, respectively, despite their high porosity. The research provided a promising approach to prepare highly porous ZTA composites with high strength for a wide range of applications.

## 1. Introduction

Porous ceramics are extensively used in a wide range of applications, including filtration (water treatment and catalysis), energy production (solid oxide fuel cells, SOFC) [1,2], and lightweight structural materials (thermal insulator, metallurgy, autoclaved aerated concrete) [3]. Porous ceramics have also attracted increasing interest in biomedical applications, such as in different implants and bone scaffolds [4,5].

Several manufacturing methods have been elaborated to produce porous structures including gel-casting process [6], organic foam technique, freeze casting [7,8], pore-forming agent method [9,10], or partial sintering by spark plasma sintering (SPS), employing appropriate sintering conditions, e.g., uniaxial pressure or graphite tool configuration [11]. Generally, each has its own merits and limitations. The polymer foam replication technique was established to acquire a morphology with relatively large, interconnected pores of several hundred microns; millimeters in terms of size. However, the ultra-high porosity, having large connections, usually results in poor mechanical properties, especially because several structural defects are formed in the course of pyrolysis of the polymer foam. Thus, porosity has great influence on the mechanical properties of bulk material [3,12], and it is a great challenge to find the optimum between porosity and enhanced mechanical properties. Consequently, simultaneously developing a reliable route to fabricate porous ceramics with high porosity and high strength has become an important task.

Porosity gradient structured ceramic materials may provide a solution for this apparent contradiction. In these ceramics, the porosity shows a continued change along the axial direction of the bulk material. Gradient porosity structures have also attracted interest in medicine for tissue regeneration, as they provide porosity with proper mechanical strength. In our previous studies, we showed a simple way to prepare a ceramics body with gradient porosity, employing asymmetric graphite configuration during SPS [11,13].

One of the promising methods for making porous ceramics is the application of ceramic fibres as raw material, which has been demonstrated in numerous papers [14,15,16]. Most of these articles reported the formation of a three-dimensional network of interconnected pores [4,17]. The mechanical properties of these connected porosity network structures are greatly influenced by the type of the applied fibres. Using chopped fibres as starting material results in lower stability of the ceramic part, since only weaker bonds develop between the overlapping fibres. In contrast, continuous fibres may provide a better choice for making porous fibrous ceramics, as the as-spun fibres already contain so-called interfibre bondings. Chavoshnejad et al. [18] showed that bonds among all crossing points of the intersecting fibres significantly increases the stiffness irrespective of material properties and loading conditions.

The mechanical stability of fibrous ceramics can be increased by applying ceramic fibre with adequate stability and mechanical properties, such as alumina or zirconia. The incorporation of 20–30 wt% ZrO_2_ in an Al_2_O_3_ matrix leads to a ceramic composite called zirconia-toughened alumina (ZTA) featuring considerably enhanced hardness [19,20,21,22] and fracture toughness [23,24] compared to pure alumina and zirconia. Both Al_2_O_3_ and ZrO_2_ ceramic fibres can be synthetized from an electrospun precursor [25] in a simple manner, but only a few papers have been published on the production of ZTA composite fibres so far [21,26,27]. ZTA nanocomposite, made by sol-gel synthesis, offers several advantages [28,29,30,31,32] such as a highly homogeneous distribution of the Al_2_O_3_ and ZrO_2_ phases compared with the conventional mixing/milling synthesis methods due to the mostly intragranular position of ZrO_2_ [32], which results in enhanced mechanical properties [29]. In addition, Al_2_O_3_ grains were able to control the grain growth of tetragonal zirconia and hindered the unexpected transformation from tetragonal to monoclinic (t→m) phase associated with grain coarsening of zirconia [22,29,33].

To stabilize ZrO_2_ in the tetragonal crystal structure and take advantage of the well-known phase transformation toughening mechanism, an additional stabilizing component is required. The most used additions are yttria and ceria; 3 mol% Y_2_O_3_-stabilized ZrO_2_ (YSZ), as well as 16 mol% CeO_2_-stabilized ZrO_2_ (CSZ) show outstanding mechanical properties [34], and both are good candidates for reinforcing material for the Al_2_O_3_ matrix with several applications [35,36,37]. Each additive, however, has some drawbacks, which makes it questionable in terms of correct/safe applications. YSZ suffers from the so-called low temperature degradation (LTD) in humid atmospheres, resulting in early t→m phase transformation that limits its application [38]. However, Y-ZTA composite offers promising results against LTD, considering the mainly intragranular position of ZrO_2_, as reported in [22,39]. In contrast, CeO_2_-stabilized ZrO_2_ shows much better LTD resistance with excellent mechanical properties. CeAl_11_O_18_ phases can also be formed during SPS due to solid-state reactions between Ce_2_O_3_ and Al_2_O_3_, as was reported in a previous study [13]. It should be noted that composite fibre with highly homogeneous distributions of Al_2_O_3_ and ZrO_2_ phases, as well as the intragranular position of ZrO_2_, are promising in avoiding adverse reactions.

The aim of this study is to demonstrate the successful synthesis of porous Al_2_O_3_ and ZTA ceramics with different additions (Y_2_O_3_, CeO_2_) using continuous composite nanofibre precursors and a fast sintering method (SPS) to create a porosity gradient structure. We investigated the effect of sintering conditions and the developed temperature distribution within the specimen during sintering on the microstructure and the mechanical features of the fabricated materials.

## 2. Materials and Methods

### 2.1. Synthesis of Ceramic Nanofibres

For the fibrous ceramic body, pure Al_2_O_3_ and CeO_2_- and ZrO_2_-stabilized ZTA composite fibres were respectively prepared by electrospinning technique.

The precursors of the solutions required for electrospinning were aluminium nitrate nonahydrate [Al(NO_3_)_3_·9H_2_O] (Szkarabeusz Kft., Budapest, Hungary, 99.0%), zirconyl (IV) chloride hydrate (ZrOCl_2_·H_2_O) (Szkarabeusz Kft., Budapest, Hungary, 99.0%), cerium (III) nitrate hexahydrate (Ce(NO_3_)_3_·6H_2_O) (Sigma-Aldrich Kft. Budapest, Hungary; 99.0%), and yttrium (III) nitrate hexahydrate (Y(NO_3_)_3_·6H_2_O) (Sigma-Aldrich Kft. Budapest, Hungary; 99.0%).

For the ZTA composite fibres, the precursor solution with an Al_2_O_3_:ZrO_2_ weight ratio of 80:20 was synthesized by dissolving the Al(NO_3_)_3_·9H_2_O and ZrOCl_2_·H_2_O salt in pure distilled H_2_O, while for Al_2_O_3_ fibres the solution contained only Al(NO_3_)_3_·9H_2_O. In addition, Ce-ZTA and Y-ZTA precursor solutions also contained Ce(NO_3_)_3_·6H_2_O and Y(NO_3_)_3_·6H_2_O, respectively, to stabilize ZrO_2_ in the tetragonal phase. The applied amounts of these additives corresponded to 16 mol % CeO_2_-stabilized ZrO_2_ and 3 mol% Y_2_O_3_-stabilized ZrO_2_.

To obtain solutions suitable for fibre spinning, poly(vinylpyrrolidone) (PVP) with a molecular weight of 1.3·10^6^ g/mol was used as auxiliary material. PVP was dissolved in ethanol (Molar Chemicals, Budapest, Hungary, 99%) at a concentration of 25 wt%. The PVP-ethanol solution was added to the Al_2_O_3_ and the ZTA precursor solutions in 1:1 ratio, and stirred at room temperature for 60 min to adjust the required viscosity for fibre spinning. The appropriate solutions were subsequently injected from a plastic syringe through a stainless steel needle with an inner diameter of 1 mm, at a constant flow rate of 15 cm^3^ h^−1^ using an Aitecs (Vilnius, Lithuania) SEP-10 syringe pump. A direct current power supply (MA2000 NT 35/P, Budapest, Hungary) set at 35 kV was connected to the needle. The nanofibers were collected on aluminium foil fixed on a metal plate. The distance was 20 cm between the tip of the needle. After the nanofibres had been removed from the aluminium foil, they were heat treated in a furnace at 1200 °C for 1 h in an atmosphere of air.

### 2.2. Synthesis of Fibrous Porous Ceramics Composites

Pure Al_2_O_3_ and both ZTA fibrous samples stabilized with different additives were consolidated with the use of a SPS machine (HPD25, FCT System GmbH, Rauenstein, Germany) under vacuum at two temperatures (1200 °C, 1300 °C) for 4 min, controlled by the set temperatures of the pyrometer of the SPS. Both the heating and cooling rate were set to 100 °C/min. The samples were sintered under a uniaxial pressure of 12 MPa in order to obtain porous structures. We employed a 12 ms on and 3 ms off pulse cycle on the SPS machine with a characteristic time of 3.3 ms for each single pulse.

**Figure 1:**
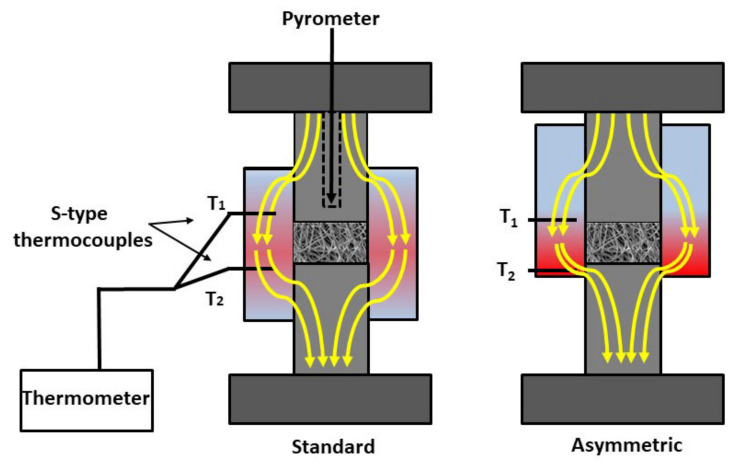
A schematic illustration of the graphite tool arrangement, as well as the sample positions in SPS.

In addition, in order to create a porosity gradient structure, we employed an asymmetric graphite configuration, in which the fibrous sample was placed in an asymmetric position (ASY) with respect to the graphite mould. This asymmetric arrangement generated a temperature gradient within the sample. As reference materials, sintering tests were also carried out in standard graphite configurations (fibrous sample placed in the middle of the graphite mould) (STD). To determine the temperature gradients in the samples, two thermocouples (S-type) were inserted into the wall of the graphite tools both at the top (T_1_) and bottom (T_2_) parts of the samples, respectively, as shown by Figure 1.

### 2.3. Characterization of Fibrous Porous Ceramics Composites

The microstructure and mechanical properties of the samples were characterized by various techniques. The relative density of the sintered specimens was determined using the Archimedes method, from which the apparent porosity of the samples was calculated.

We analysed the phase composition and determined structural characteristics of the as-prepared samples by XRD analysis (Philips PW 1830) using Cu Kα radiation. The XRD measurements were carried out in the range of 15–70° 2θ in step-scanning mode, with a step length of 0.04° for 1 s acquisition time per angle. This analysis was performed on the as-synthetized ceramic fibres and on both sides of the sintered ceramic bodies.

The cross-section of the fractured samples was analysed by SEM (Zeiss EVO40, Zeiss, Germany) with the use of a secondary electron detector (SE) operated at 20 kV at various magnifications. The samples were also examined by a backscattered electron (BSE) detector; the images were acquired at 3 and 10 kV using SEM (JSM-IT700HR, JEOL,Tokio Japan). In terms of mechanical features, hardness and compressive strength were determined.

Vickers hardness measurements were carried out by depth-sensing micro-indentation tests using a CSM2008 instrument (Peseux, Switzerland) provided with a Vickers indenter tip. The surfaces of the top and bottom sides of the samples were cut and polished prior to the tests. During each measurement, a load was held for 15 s with a force of 983 mN. Hardness was given as the mean value of five indentation tests performed on the top and bottom parts of the ceramic bodies. For each indentation test the load–penetration depth curve was automatically plotted. The Vickers hardness was calculated from the unloaded area of the load–depth curve.

Uniaxial compression testing was conducted at room temperature using an Instron 5566 tester applying a constant cross-head speed of 0.5 mm/min. Strength measurements (on three specimens for each specimen type) were performed on the specimens with 7 mm × 7 mm × ~4 mm dimensions.

## 3. Results and Discussion

### 3.1. Characterization of the Ceramic Nanofibres

**Figure 2:**
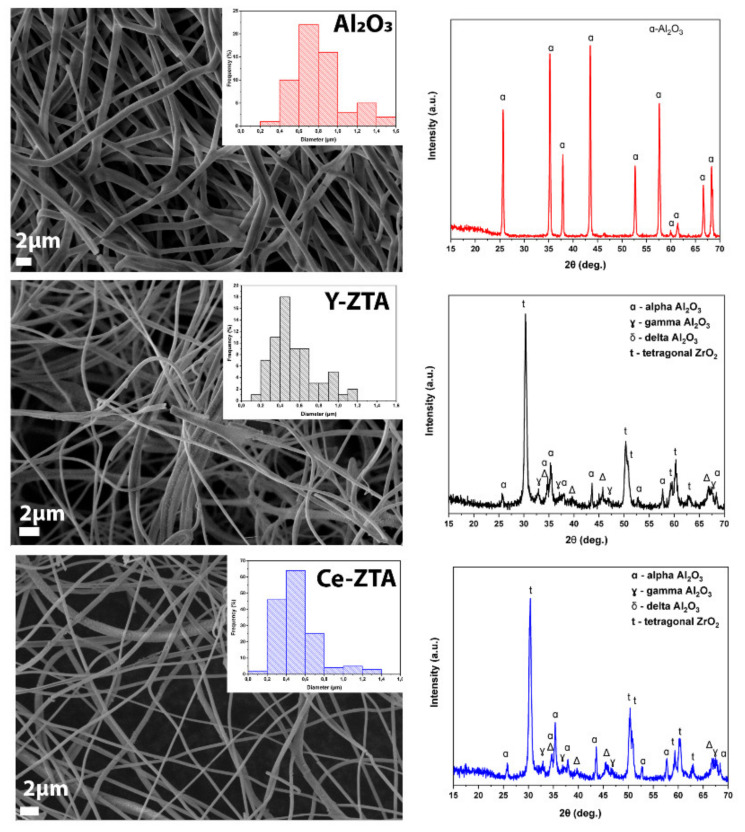
Characterisation of reference fibres by SEM and XRD analysis methods.

The SEM and XRD results of the initial ceramic fibres are shown in Figure 2. The phase composition of the fibres is not uniform even when all three types of precursor fibres are calcinated with the same conditions. This phenomenon is obviously explained by the presence of ZrO_2_. Based on previous observation by [30], the ZrO_2_ content retards the stable α-alumina transformation. In the pure Al_2_O_3_, without any ZrO_2_ addition, only the characteristic peaks of α -Al_2_O_3_ can be detected. However, ZTA composite fibres consist of two different phases of Al_2_O_3_ beside the tetragonal ZrO_2_, fitted by JCPDF 81-1544 reference. The peaks of alumina belong to γ-; δ- and α–Al_2_O_3_ phases with the reference of JCPDF 01-1308, 16-394, and 10-0173, respectively, for both Ce- and Y-ZTA fibres. The average fibre diameters were 800 ± 21 nm for Al_2_O_3_, whereas they were 530 ± 23 and 520 ± 21 nm for Y-ZTA and Ce-ZTA composite fibres, respectively, which were determined by SEM image analysis. The distribution of fibre diameters is also shown in the right corner of the SEM images (Figure 2).

### 3.2. Temperature Distribution Monitoring during the SPS Heat Treatment

**Figure 3:**
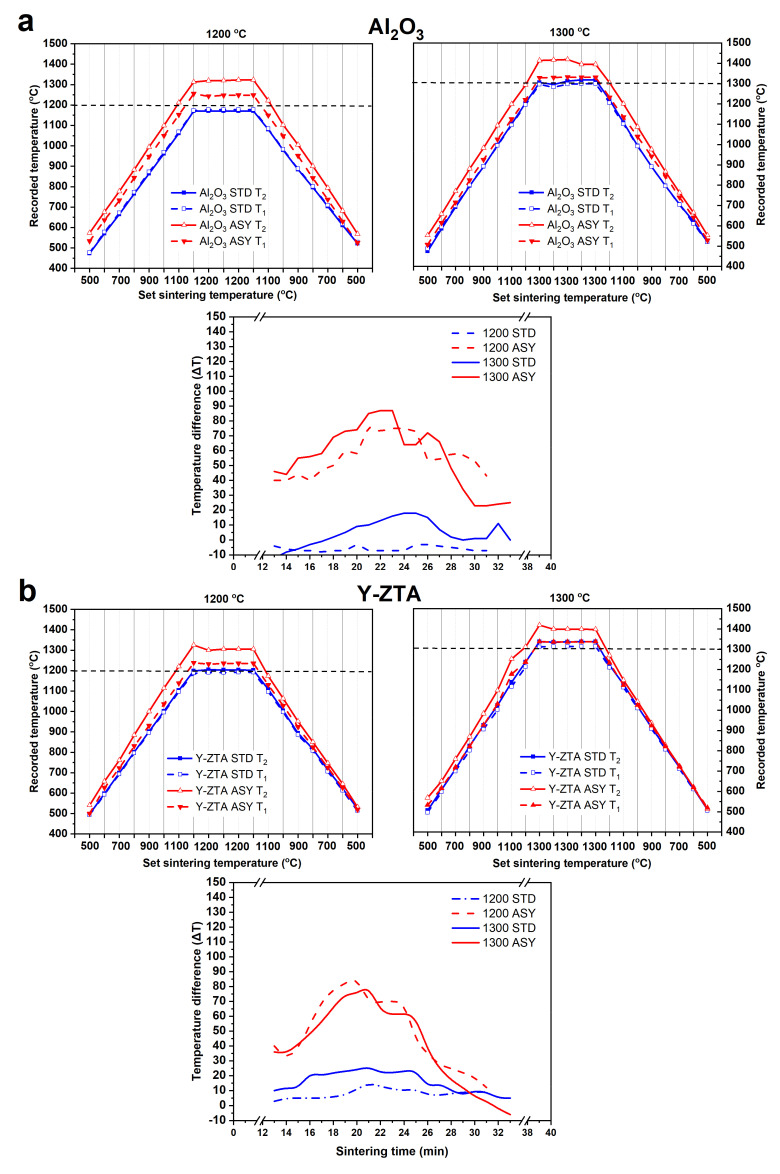

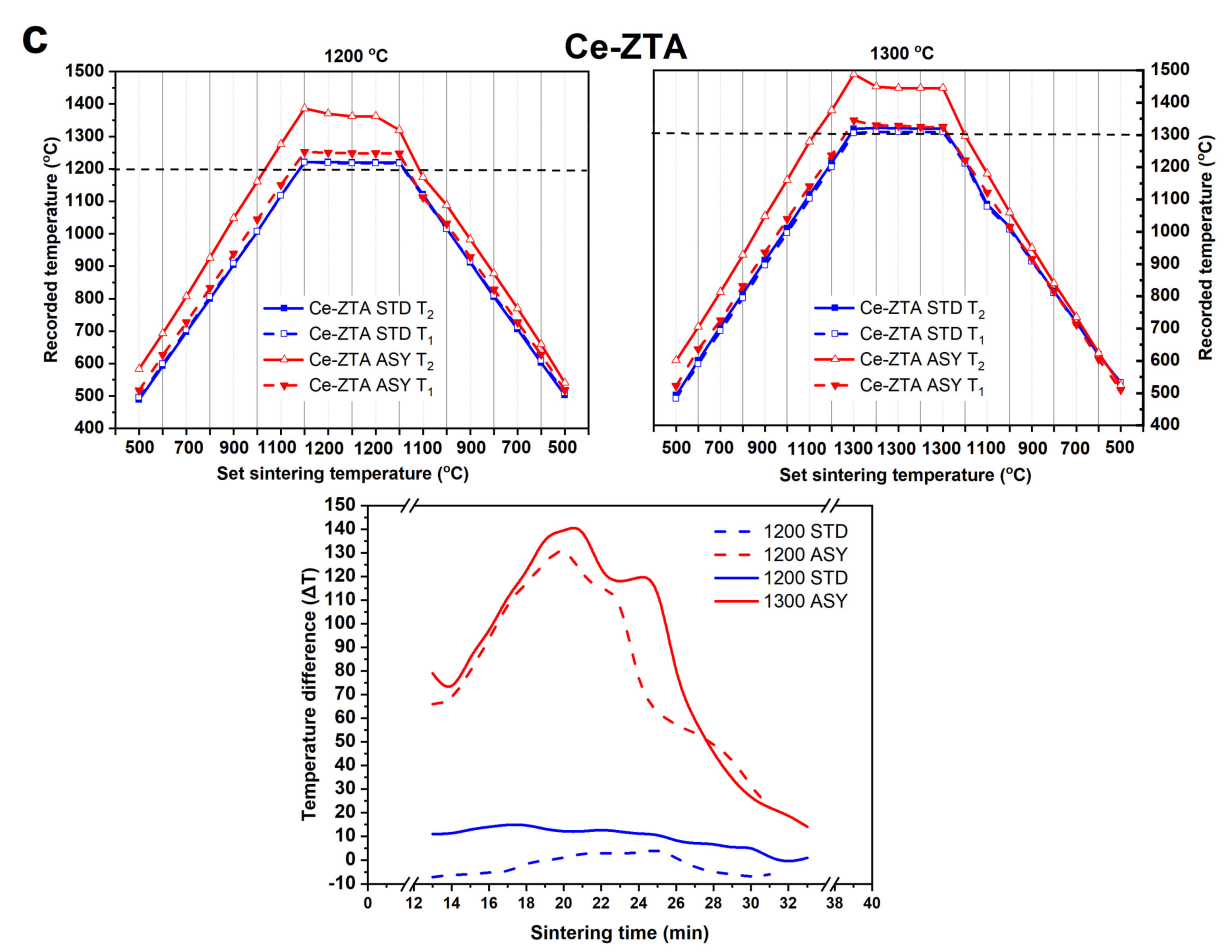
The top (T_1_; full symbol) and bottom (T_2_; empty symbol) temperatures of (**a**) Al_2_O_3_, (**b**) Y-ZTA, and (**c**) Ce-ZTA samples recorded in STD (blue line) and ASY (red line) at both sintering temperatures are shown as a function of the set temperature, measured by pyrometer (horizontal dash line illustrates the set temperatures). The calculated ∆T with respect to sintering time for ASY and STD configurations are also shown.

Gradient microstructures were successfully developed in the fibrous ceramic body by establishing a temperature gradient during sintering. The temperature difference is established between the top and bottom sides of the samples during the SPS sintering process applying an asymmetric graphite configuration, similar to that reported in our previous studies [11,13]. In Figure 3, we show the recorded temperature at the top (T_1_) and bottom side (T_2_) of the sample as a function of set temperatures during the sintering process in the STD and ASY series. These figures also illustrate the temperature difference (∆T) between T_1_ and T_2_ (T_2_ − T_1_ = ΔT) with respect to sintering time for the series.

It can be clearly seen from the temperature data (Figure 3) that temperature values are almost equal between the opposite sides of the samples at 1200 °C for STD samples. With increasing sintering temperature, the ΔT reached only a few degrees (max. 20 °C). In contrast, ΔT approached or even exceeded 100 °C for the ASY series regarding the various compositions; in alignment with our expectations. The bottom side of the samples had a typically higher temperature than the top side. Notably, the largest ΔT occurred always in the heating period and gradually decreased as the sintering progressed, the possible reasons for which we have discussed in our previous studies [11,13]. The temperature difference between the top and bottom sides also varied with sintering set temperature: it increased with the sintering temperature, reaching a max. value of ca. 80 °C for the Al_2_O_3_ and the Y-ZTA, and 143 °C for the Ce-ZTA at 1300 °C. As Figure 3 clearly shows, ΔT between the opposite sides also varied with respect to the composition of the samples. The highest local temperatures and ΔT was recorded for Ce-ZTA (Figure 3c). The actual temperatures for this sample were 1345 °C and 1488 °C at the top and bottom sides of the samples, respectively, when applying 1300 °C sintering temperature. This ΔT corresponded to a temperature gradient of ca. 44 °C/mm, which anticipated the formation of a significant gradient in the microstructure of the bulk sample. For the Y-ZTA and Al_2_O_3_ samples, ca. 25 °C/mm and 20 °C/mm high temperature gradients were established, respectively, considering the thickness of bulk samples and the recorded ΔT.

### 3.3. Microstructure of the Fibrous Composites

**Figure 4:**
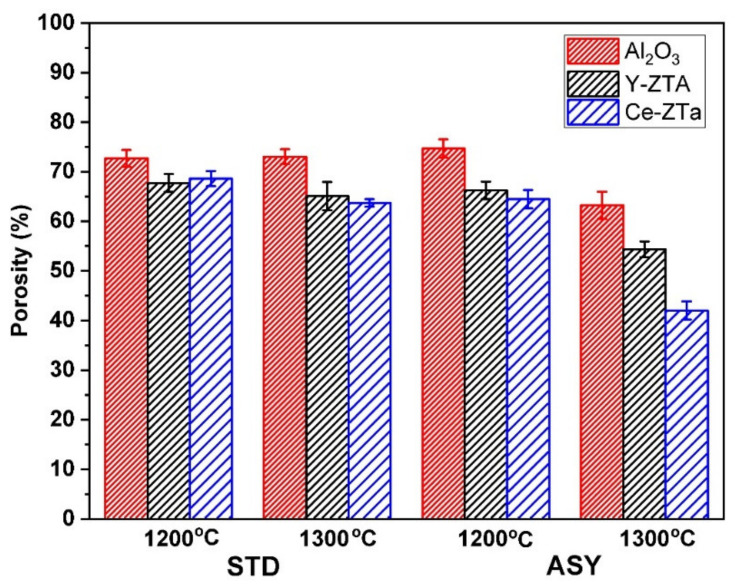
Apparent porosity of the ceramic bodies manufactured at different temperatures in ASY and STD graphite configurations. Red, black, and blue bars represent the samples of Al_2_O_3_, Y-ZTA, and Ce-ZTA, respectively.

The apparent porosity of the Al_2_O_3_, Y-ZTA, and Ce-ZTA composites sintered at various temperatures are shown in Figure 4.

Relatively high porosity was attained for all the fibrous samples in the applied sintering conditions. The highest porosity was obtained for pure Al_2_O_3_ samples regardless of the sintering conditions, which can be explained by several factors. The melting point of ZTA is lower than that of pure Al_2_O_3_; thus, the ZrO_2_ addition increases the bonding between them and ultimately promotes higher density. The high ΔT generated in ZTA composites also contributes to greater shrinkage, leading to lower porosity of these samples. Furthermore, γ → δ → α- Al_2_O_3_ transformation also occurs in the ZTA composites during sintering, which reduces the porosity of the samples, causing a decrease in volume [40].

The porosity varied between 75 and 65% for samples sintered at 1200 °C. At this temperature, the graphite configuration had a negligible influence on porosity. At 1300 °C, however, noticeable differences could be observed in the densities, especially for ASY, where the local temperature was significantly higher at the bottom (Figure 3). While the porosity of the samples was as high as 65 and 63% in the STD configuration for the Y-ZTA and Ce-ZTA samples, respectively, it dropped to 54 and 42% in the ASY conditions because the local temperature approached 1400 °C and 1500 °C at the bottom side of these samples.

**Figure 5:**
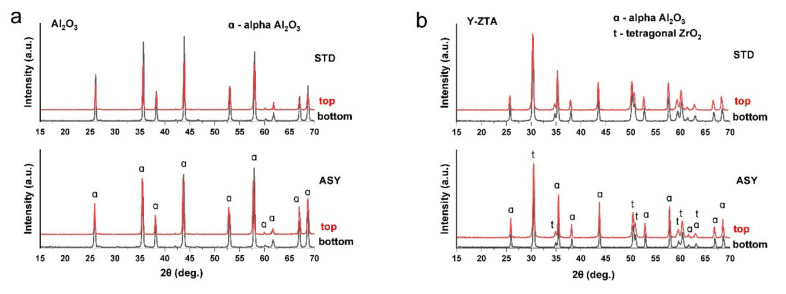

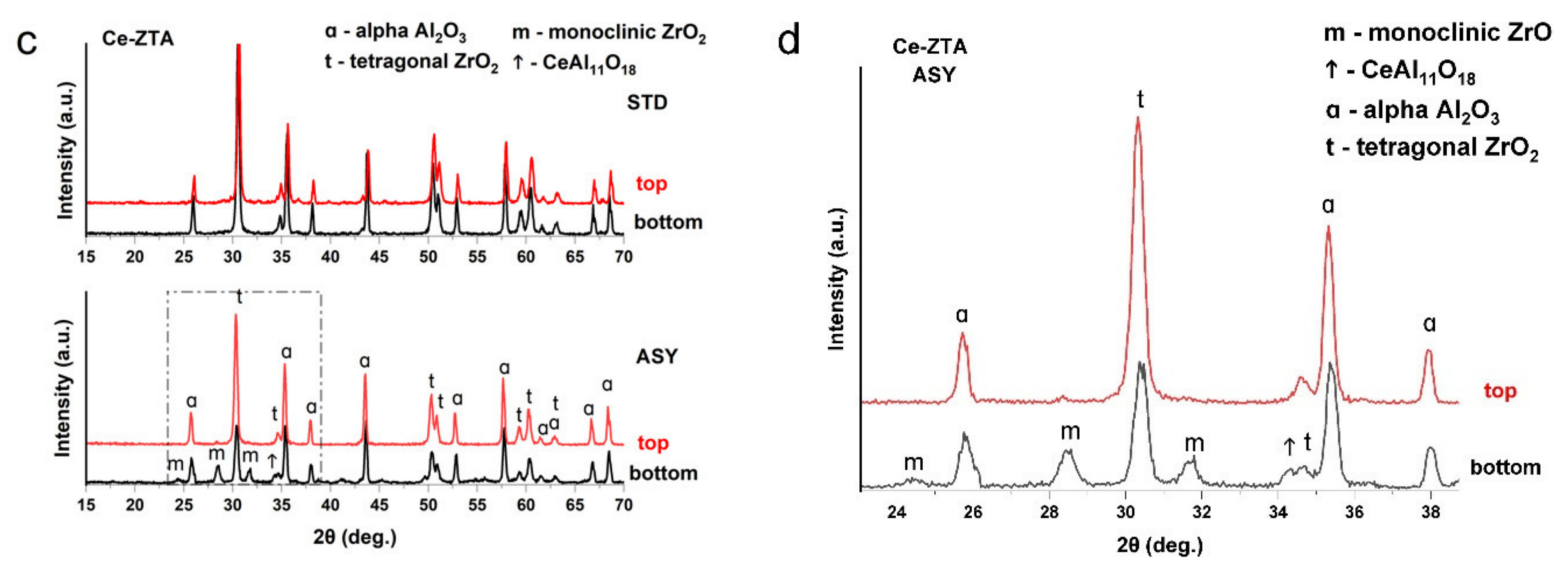
Phase composition of (**a**) Al_2_O_3_, (**b**) Y-ZTA, and (**c**) Ce-ZTA samples fabricated in STD and ASY modes at 1300 °C. (**d**) Enlarged XRD pattern of the Ce-ZTA sample fabricated in ASY focusing on the phase differences between the top and bottom side.

The phase compositions of sintered ceramic bodies were determined by XRD analysis. We performed the analysis on both top and bottom sides of the sintered samples to observe the effect of the temperature differences on phase composition. The detected phases of the initial and sintered samples are summarized in Table 1. Figure 5 represents the XRD patterns of the pure Al_2_O_3_ and of both ZTA samples fabricated in STD and ASY arrangements at a 1300 °C sintering temperature.

As Table 1 shows, in STD configurations at both sintering temperatures, the phase composition is similar on both sides of the sample disc. Significant differences could not be observed, regardless of the sintering temperature; only α-Al_2_O_3_ and t-ZrO_2_ were detected. Thus, the initial nanofibres containing γ- and δ- Al_2_O_3_ polymorph phases were transformed into α form. Surprisingly, neither CeAl_11_O_18_ nor m- ZrO_2_ could be identified in the samples (Figure 5a–c), which confirmed our assumption that a sol-gel synthesis method resulted in homogeneous distribution of the components; therefore, the formation of the unexpected abovementioned phases can be avoided.

In the ASY series, the phase composition was the same as in STD series for the Al_2_O_3_ and Y-ZTA samples at sintering temperatures of 1200 °C and 1300 °C. Between the two sides of these samples, there were differences only in respect to the average crystal sizes. As the temperature increased at the bottom, the crystal size increased, suggested by the slight broadening of the peaks. In contrast, for the Ce-ZTA samples, different phase compositions were detected on the top and bottom sides of the samples at both sintering temperatures (Figure 5c,d). We recorded the highest local temperatures in this sample, especially at the bottom, leading to different phase compositions on the two sides, as shown in the enlarged figure (Figure 5d). As anticipated, CeAl_11_O_18_ appeared among the phases, however, only at the bottom side of Ce-ZTA sintered at either 1200 °C or 1300 °C. The high temperature gradient also affected the t→m phase transformation of ZrO_2_ in the Ce-ZTA samples. On the top side, t-ZrO_2_ and α-Al_2_O_3_ were detected, and the m-ZrO_2_ phase appeared on the opposite side at both sintering temperatures. In the sample sintered at 1200 °C, the m-ZrO_2_ was identified in very small amounts; however, the amounts increased with increasing sintering temperatures. Nevertheless, the main phase remained t-ZrO_2_. The m-ZrO_2_ that appeared is related to the increasing temperature along the samples during the sintering process, reaching a critical grain size of ZrO_2_ (<0.4 µm), which induces spontaneous and partial t→m transformation [32,41]. Compared to the results of both ZTA samples regarding the phase transformation of ZrO_2_, it seems that this phase transformation can be avoided for Y-ZTA. Thus, we can conclude that Y_2_O_3_ addition renders better stabilization for t-ZrO_2_ than CeO_2_ under these SPS sintering conditions. However, the applied sol-gel synthesis process for Ce-ZTA could also be a promising way to avoid or highly reduce the reactions between CeO_2_ and Al_2_O_3_ during SPS at proper sintering temperatures.

Al_2_O_3_, Y-ZTA, and Ce-ZTA composites were analysed by SEM technique to determine the effects of graphite configurations on the microstructure of the fibrous ceramic bodies. The microstructure investigation focused on the cross-section of the fractured surfaces of the top and bottom parts of the samples. As was expected, different microstructures were observed in the samples with respect to the applied sintering conditions (temperature, graphite configuration) and the sample composition. According to the applied STD or ASY configuration, the highly porous frame structure of the ceramic fibres was well-reflected in the structure of the sintered specimens.

**Figure 6:**
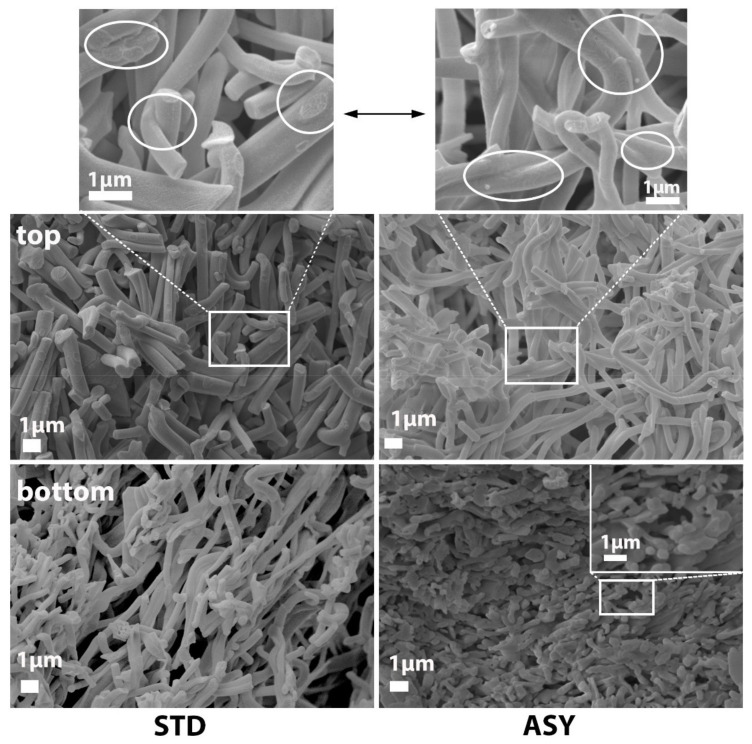
Comparative SEM micrographs of the (**top**) and (**bottom**) fractured surfaces of the Al_2_O_3_ samples fabricated in STD and ASY graphite arrangements at a 1300 °C sintering temperature. White circles on the high magnification images show the contact areas of the fibres.

**Figure 7:**
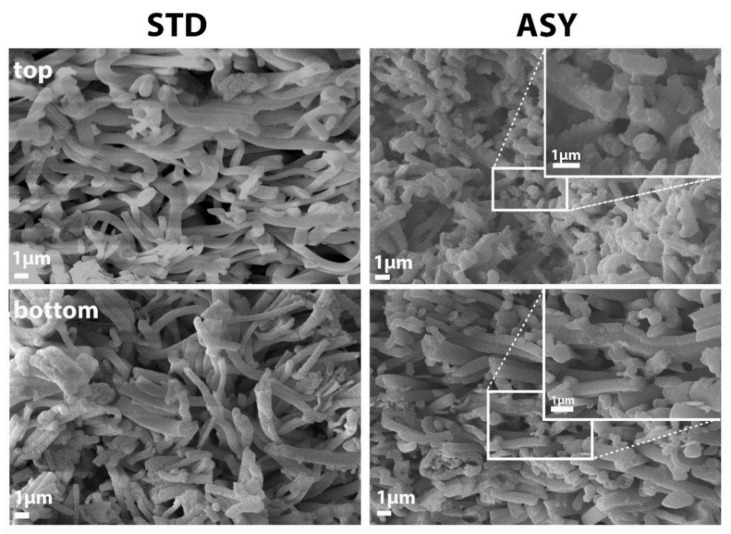
Comparative SEM micrographs of the (**top**) and (**bottom**) fractured surfaces of Y-ZTA samples fabricated in STD and ASY graphite arrangements at a 1300 °C sintering temperature.

**Figure 8:**
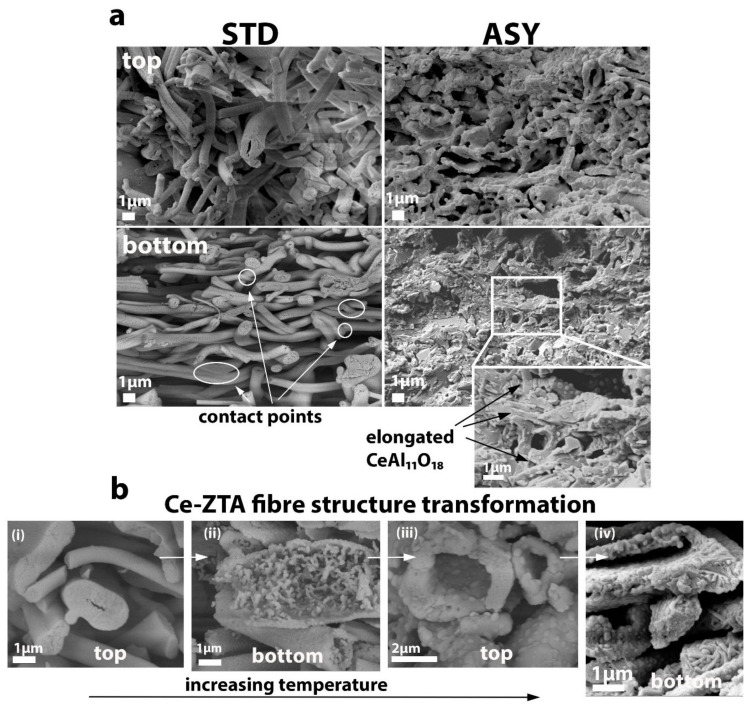
Comparative SEM micrographs of (**a**) the top and bottom sides of the fractured surface of the CE-ZTA samples fabricated in an STD and ASY graphite arrangement at a 1300 °C sintering temperature, and (**b**) the Ce-ZTA fibre structure transformations fabricated in ASY; (i) and (ii) are sintered at 1200 °C, whereas (iii) and (iv) are at 1300 °C.

The sintering temperature of 1200 °C was less effective, since a weakly bonded fibrous structure was created. The fibres were randomly arranged and simply stacked without extensive bonds between them at the crossing points, particularly for the STD samples. In these samples, without the formation of a gradient temperature, the microstructure was homogeneous through the cross-section of the bodies. In contrast, gradient microstructure was revealed for the ASY samples, while the structure in the top parts contained only a few connection points; the stronger frame structure was formed in the bottom parts. The structural changes, e.g., the effect of STD or ASY arrangement on the microstructure, were more obvious in the samples sintered at 1300 °C, shown by Figure 6, Figure 7 and Figure 8. By raising the sintering temperature to 1300 °C, structures became denser and, therefore, stronger due to a noticeable neck formation between the Al_2_O_3_ fibres, as shown by the images in Figure 6. However, in the case of the STD sample, the structure was still fragile and the fibres looked fragmented, considering the limited extension of the neck formation and lack of further progress in neck growing, as well as in the contacted areas (highlighted by white circles on the high magnification image). In contrast, the ASY configuration exhibited a gradually increasing density in the direction of the bottom section of the Al_2_O_3_ sample, leading to reduced porosity in that region. In contrast, the microstructure of the top part of the samples preserved their highly porous features (Figure S1).

In addition to the higher sintering temperatures, the addition of ZrO_2_ also assists in improving the structural stability of ceramics. ZrO_2_ contributes to intensive neck formation at the contact points, resulting in increasing fibre connection areas and stronger frame structures in the Y-ZTA (Figure 7) and Ce-ZTA (Figure 8) samples, as compared with the pure Al_2_O_3_ sample (Figure 6). This observation is also supported by the decreased porosity results.

However, the most obvious changes can be observed in the cross-section of the Ce-ZTA sample comparing the STD and ASY samples. As Figure 8 shows, on the top sides of the Ce-ZTA samples sintered in STD, the fibrous structure is randomly oriented, containing several connected fibres. In contrast, the top side of the ASY sample has a stabilized frame structure with an extensive connected porosity network. As the temperature gradually increases towards the bottom side, the contact areas at the crossing of the fibres significantly increase, reducing the porosity in parallel (Figure S3). The graded structural alteration (especially in the porosity) is in direct relationship to the recorded ∆T in the sintered body. Accordingly, a less significant difference is detected in the Al_2_O_3_ and Y-ZTA samples, as shown in Figures S1 and S2, while it is relatively significant for the Ce-ZTA samples, particularly for the sample sintered at 1300 °C, where the highest ∆T (143 °C) was recorded. The SEM examinations revealed elongated CeAl_11_O_18_ crystals in the microstructure of the Ce-ZTA samples (Figure 8a and Figure S3).

Significant structural changes can also be detected by comparing the morphology of the initial and sintered fibres. Regardless of the sintering configurations or the composition of the fibres, they all acquired a hollow structure after sintering. The exact mechanism has not been completely revealed; however, we suppose it can be attributed to the Ostwald-ripening mechanism, which is responsible for the formation of several hollow structures, including fibres [42,43]. Through SEM examinations, the hollow formation of the fibres can be seen in the Ce-ZTA samples fabricated in ASY at 1200 °C and 1300 °C. As Figure 8b shows, at 1200 °C, the hollowing process of fibre had already started in the microstructure on the top side (i), but it had not completely taken place, and the bottom part of the inside fibre showed a highly porous structure (ii). When the set sintering temperature increased to 1300 °C, the process was completed, and the fibres became entirely hollow with a dense shell at the top side of the sample (iii). However, as can be observed, due to the relatively high temperature developed on the bottom side, the hollow fibres collapsed and flattened (iv). The hollow fibres also affected the total porosity of the bodies. On one hand, the composites comprised large pores of micrometre sizes formed among the interconnecting fibres, and on the other hand, nanometre-sized pores within the hollow fibres. In brief, bimodal porosity could be identified within the samples.

**Figure 9:**
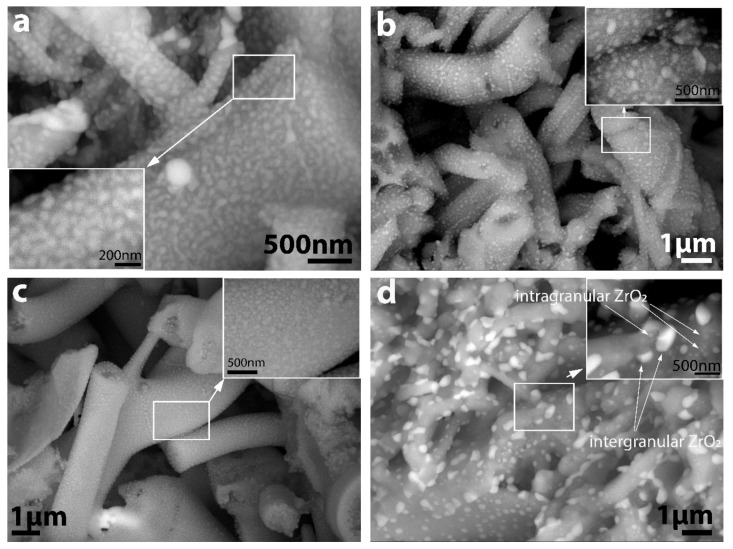
SEM-BSE micrographs of the fractured surface of (**a**) Y-ZTA STD, (**b**) Y-ZTA ASY, (**c**) Ce-ZTA STD, and (**d**) Ce-ZTA ASY at a 1300 °C sintering temperature.

We performed SEM analysis to reveal the detailed microstructure of the composite fibres with special attention given to the location and distribution of ZrO_2_ in the Al_2_O_3_ matrix. Heuer et al. [32] found ZrO_2_ particles were located in intragranular positions inside the Al_2_O_3_ particles in the ZTA composite processed by a similar sol-gel method. SEM-BSE images (Figure 9) provided evidence that the ZTA fibres were composed of a binary phase microstructure of Al_2_O_3_ and ZrO_2_, and their distribution was highly uniform, especially for STD series for both types of ZTA fibres, where nanostructured ZrO_2_ grains exclusively occurred (in the figures, the grey particles represent the Al_2_O_3_, and the white particles correspond to ZrO_2_.) However, high magnification images (Figure 9b, d) show that the ZrO_2_ particles were present in two different sizes located in different positions in the ZTA composite fibres sintered in ASY conditions. The large grains were in intergranular positions at the grain boundaries of the Al_2_O_3_, assuming they were formed by the coalescence of small ZrO_2_ grains at the high sintering temperature, supposedly due to the abovementioned Ostwald-ripening mechanism. The small grains remained in the original, intragranular positions within the Al_2_O_3_ matrix. Thus, an intra/inter-type nanocomposite structure was developed. The final size of the larger zirconia grains varied, depending on the doping elements; the size of zirconia grains in the Ce-ZTA samples was twice as much as in the Y-ZTA samples. This difference can probably be accounted for by the higher top values of temperature that occurred during sintering. In contrast, the abovementioned bimodal distribution of zirconia could not be observed on the fibres sintered by STD configurations (Figure 9a,c).

### 3.4. Mechanical Behaviour of the Composites

The mechanical properties of the fibrous composites were characterised in terms of microhardness and compression strength.

**Figure 10:**
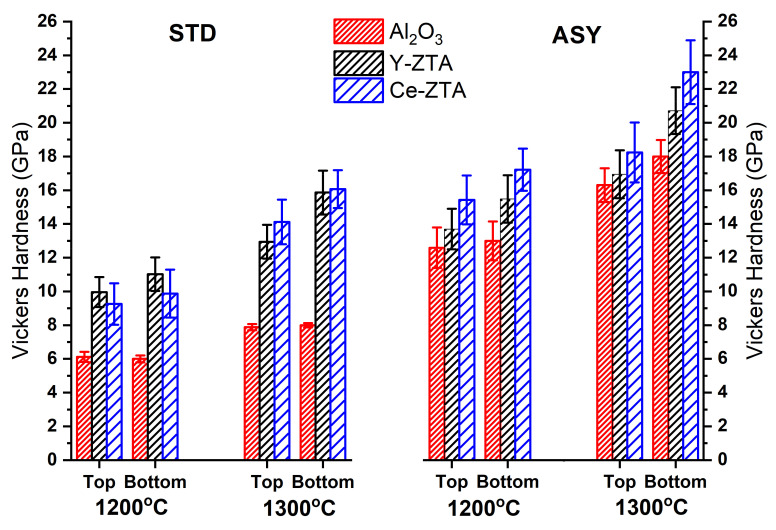
Vickers hardness of the samples fabricated in ASY and STD configurations at 1200 °C and 1300 °C set sintering temperatures.

Figure 10 shows the mean hardness values of the STD, as well as the ASY series. We performed Vickers indentation tests on both sides of the samples to see the effect of the gradient microstructure developed along the cross-section in the ASY samples. Generally, the hardness was closely related to the porosity of the materials, but no univocal correlation could be provided in a complex system having gradient microstructure and bimodal porosity (micro- and macroporosity), as revealed by the SEM investigations.

As shown in Figure 10, the Al_2_O_3_ samples sintered at 1200 °C exhibited the lowest hardness (6.1 ± 0.3 GPa) that is in line with the microstructure investigations; this sample exhibited the highest porosity and the smallest contact area among the fibres. At a higher sintering temperature of 1300 °C, only a small improvement can be observed in the hardness (8.0 ± 0.2 GPa). In contrast, Y-ZTA and Ce-ZTA composites show a twofold increase (15.8 ±1.3 and 16.1 ± 1.2 GPa, respectively), suggesting that ZrO_2_ addition promotes the compaction of the fibrous ceramics. Considering the negligible ∆T recorded between the opposite sides of the samples at both sintering temperatures in the STD arrangement, the difference in the measured microhardness values is also small between the opposite sides.

Regarding values found in the literature, the obtained HV values are still roughly the same as those published in the literature [28,44], even though our samples possess higher porosity.

In contrast, the effect of the ASY arrangement and the accompanied higher local temperature is clearly reflected by the HV of the samples. It can be concluded that the application of ASY configuration leads to increasing hardness for all the samples. Furthermore, the top and bottom HV values consider the gradual porosity changes of the composites, since the HV is always lower at the top side than at the opposite side of the ceramic bodies. Similarly, to the STD series, the Al_2_O_3_ samples show the lowest HV values followed by the Y-ZTA and the Ce-ZTA, respectively, which is consistent with the porosity tendency.

The HV values of Al_2_O_3_ and Y-ZTA sintered at 1200 °C in ASY and the samples made in STD configurations at 1300 °C are almost equal, related to the very similar recorded temperatures. In the STD samples, the maximum temperatures are similar to the set temperatures (around 1300 °C). For the ASY samples, we detected relatively similar temperatures (1313 °C and 1301 °C) on the bottom side for Al_2_O_3_ and Y-ZTA, respectively, despite the set temperature being 1200 °C. As a consequence of the higher sintering temperatures, the HV values of these samples also increase, and the differences become more significant between the two sides, according to the gradient microstructure. Regarding the sintering conditions and the compositions, the highest HV values were achieved for these samples. The HV is 18.2 ± 1.7 and 23.0 ± 1.9 GPa at the top and bottom sides, respectively, for the Ce-ZTA composite sintered by a 1300 °C set temperature, where the temperatures approached ca. 1500 °C at the bottom side; the total porosity of the sample was only 42 ± 2%. Considering the high porosity of the samples, it can be concluded that the achieved HV values are high compared to the other research findings [20].

**Figure 11:**
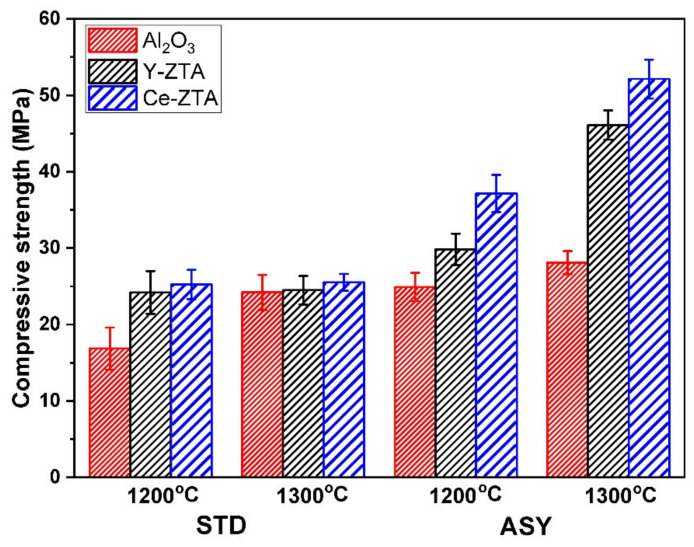
Compressive strength of the samples fabricated in ASY and STD configurations at 1200 °C and 1300 °C set sintering temperatures.

Figure 11 shows the compressive strength of porous fibrous ceramics in terms of sintering temperature and graphite configuration. The compressive strength of the samples increased with higher sintering temperature, especially for the sample sintered in ASY configuration. This can also be attributed to the strong correlations between the compressive strength and the microstructure, in a similar way to microhardness.

At a 1200 °C sintering temperature, the composite densification was rather restricted, which resulted in low compressive strength, particularly for STD samples. Having similar high porosity, the compressive strength of samples in ASY configuration invariably increased, supposing that the stronger frame structure developed at the bottom side enhanced the overall strength. Similar trends can be observed for the samples sintered at 1300 °C in both STD and ASY configurations.

As was also visible during the SEM examinations, ZrO_2_ additions enhanced structural stability and, therefore, the compressive strength of the composites, similarly to hardness. Thus, ZTA composites exhibited higher strength than pure Al_2_O_3_, as is shown in Figure 11. Consequently, in addition to the microstructure (porosity), the composition of the samples has obvious effects on the ultimate compressive strength of the samples. According to the reported studies [45,46], the intragranular ZrO_2_ effectively improves the strength of the composite, due to intragranular ZrO_2_ creating sub-grain boundaries in the Al_2_O_3_ particles; therefore, achieving refinement of the strengthening structure.

The highest strengths were reached in the Ce-ZTA and Y-ZTA samples sintered at 1300 °C in ASY configuration with the values of 52.1 ± 2.5 and 46.1 ± 1.92 MPa, respectively, even though these samples had relatively high total porosity (42 ± 2% and 54 ± 1% for Ce-ZTA and Y-ZTA sample, respectively). Several works have reported that high porosity of the composites leads to a drastically reduced compression strength of Al_2_O_3_ [1,9,12,14,47,48]. In contrast, in the present work, the fibrous Al_2_O_3_ and ZTA composites with high porosity showed a relatively high strength, which was attributed to the abovementioned gradient microstructure developed during sintering.

## 4. Conclusions

In this paper, we demonstrated a novel method for fabricating highly porous ceramic composites with improved mechanical properties. Unlike in earlier studies, nanofibres of various ceramic composites (Y-ZTA, Ce-ZTA, as well as Al_2_O_3_) were used to fabricate a ceramic composite with connected porosity networks. In addition, gradient structure was established by creating a temperature gradient in the material during sintering by employing asymmetric SPS graphite tool arrangements. The major findings can be summarized as follows:

- ASY configuration of the graphite tools resulted in considerable temperature differences on the opposite sides of the samples. The value of the difference increased with increasing sintering temperature, and its peak value also depended on the sintered material. It was the lowest (80 °C) for the Al_2_O_3_ and Y-ZTA samples, while the highest (143 °C) was for the Ce-ZTA sample at a sintering temperature of 1300 °C.
- The prevailing temperature difference between the opposite sides of the samples yielded gradient microstructures in terms of porosity confirmed by SEM analysis. The apparent porosity of the samples varied between 42 and 75%, which was also influenced by the as-formed hollow characteristics of the fibres.
- The phase composition of the Al_2_O_3_ and Y-ZTA samples did not change after sintering, the samples contained only α- Al_2_O_3_ and t- ZrO_2_, regardless of the graphite composition; however, minor grain-coarsening could be detected due to the high temperatures. For Ce-ZTA samples, the higher temperature gradient induced a moderate t→m-ZrO_2_ phase transformation, as well as the formation of CeAl_11_O_18_ in minor amounts detected at the higher temperature (bottom) side of the sample. More extensive phase transformations were hindered by the intragranular position of ZrO_2_ within Al_2_O_3_ grains.
- Despite the high porosity of the bodies, the developed gradient structure resulted in improved mechanical properties in terms of hardness and compression strength. The addition of ZrO_2_ also led to an increase in these properties.
- The highest HV, 18.2 ± 1.7 and 23.0 ± 1.9 GPa at the top and bottom sides of the sample, respectively, was obtained for the Ce-ZTA composite sintered in ASY with a set temperature of 1300 °C.
- The compressive strength of the composites reached 46.1 ± 1.9 MPa and 52.1 ± 2.5 MPa for Y-ZTA and Ce-ZTA, respectively, despite the relatively high porosity of the fibrous ZTA composites.

According to the obtained results, the present research provided a promising approach to prepare highly porous ZTA composites with high strength for a wide range of applications.

## Figures and Tables

**Table 1:** Phase composition of the samples.

Sample	Sintering Conditions		Sample Site	Phase Composition					
				Al_2_O_3_			t-ZrO_2_	m-ZrO_2_	CeAl_11_O_18_
				γ	δ	α			
Al_2_O_3_	Initial Nanofibre					x			
	1200 °C	STD	top			x			
			bottom			x			
		ASY	top			x			
			bottom			x			
	1300 °C	STD	top			x			
			bottom			x			
		ASY	top			x			
			bottom			x			
Y-ZTA	initial nanofibre			x	x	x	x		
	1200 °C	STD	top			x	x		
			bottom			x	x		
		ASY	top			x	x		
			bottom			x	x		
	1300 °C	STD	top			x	x		
			bottom			x	x		
		ASY	top			x	x		
			bottom			x	x		
Ce-ZTA	initial nanofibre			x	x	x	x		
	1200 °C	STD	top			x	x		
			bottom			x	x		
		ASY	top			x	x		
			bottom			x	x	x	x
	1300 °C	STD	top			x	x		
			bottom			x	x		
		ASY	top			x	x		
			bottom			x	x	x	x

## Data Availability

The data that support the findings of this study are available from the corresponding author.

## Supplementary Materials

The following supporting information can be downloaded at: https://www.mdpi.com/article/10.3390/nano12234165/s1, Figure S1: SEM micrographs of the fractured surface of the cross-section of Al2O3 samples fabricated in ASY graphite arrangement at 1300 °C sintering temperature. Figure S2: SEM micrographs of the fractured surface of the cross-section of Y-ZTA samples fabricated in ASY graphite arrangement at 1300 °C sintering temperature. Figure S3: SEM micrographs of the fractured surface of the cross-section of Ce-ZTA samples fabricated in ASY graphite arrangement at 1300 °C sintering temperature.
