# Supplementary material for: Preparation and Characterization of Fibrous Alumina and Zirconia Toughened Alumina Ceramics with Gradient Porosity
Source: Nanomaterials (Basel). 2022 Nov 24;12(23):4165. doi: 10.3390/nano12234165 (PMC9736005; doi:10.3390/nano12234165)
Supplement: Supplementary file 1 [file nanomaterials-12-04165-s001.zip › nanomaterials-2057647-supplementary.pdf]

Supplementary Material

Preparation and Characterization of Fibrous Alumina and Zirconia Toughened Alumina Ceramics with Gradient Porosity

Eszter Bódis [1,*], Kolos Molnár [2,3], János Móczó[1] and Zoltán Károly [1]

[1] Institute of Materials and Environmental Chemistry, Research Centre for Natural Sciences, H-1117 Budapest, Hungary

[2] Department of Polymer Engineering, Faculty of Mechanical Engineering Budapest, University of Technology and Economics, H-1111 Budapest, Hungary

[3] ELKH-BME Research Group for Composite Science and Technology, H-1111 Budapest, Hungary

* Correspondence: bodis.eszter@ttk.hu.

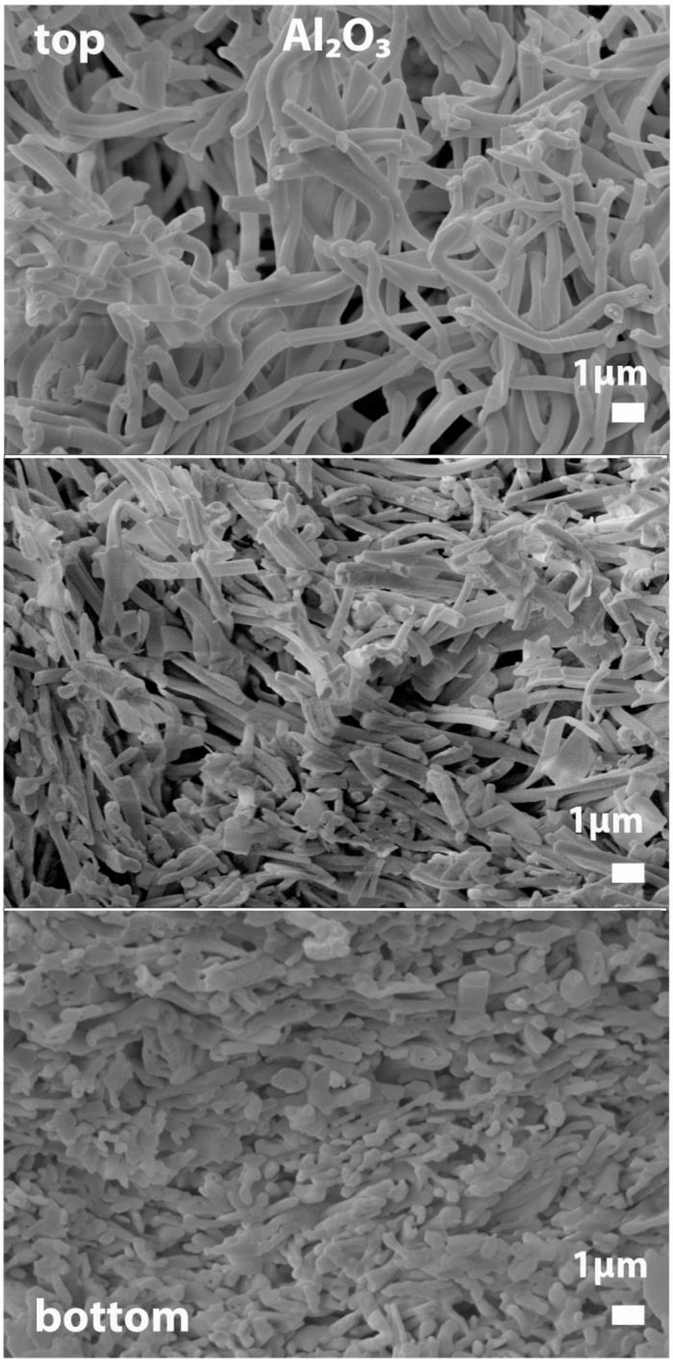

Figure S1. SEM micrographs of the fractured surface of the cross-section of Al_2O_3 samples fabricated in ASY graphite arrangement at 1300 ºC sintering temperature

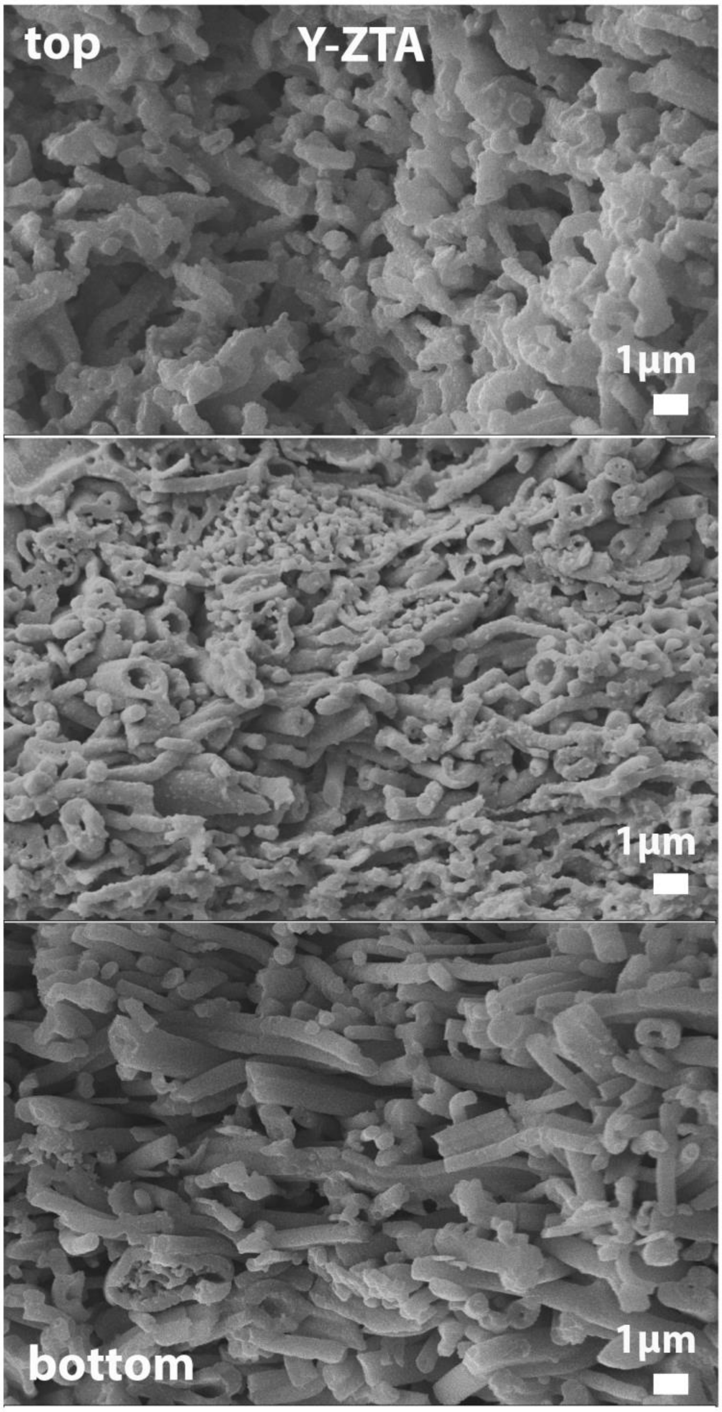

Figure S2. SEM micrographs of the fractured surface of the cross-section of Y-ZTA samples fabricated in ASY graphite arrangement at 1300 ºC sintering temperature

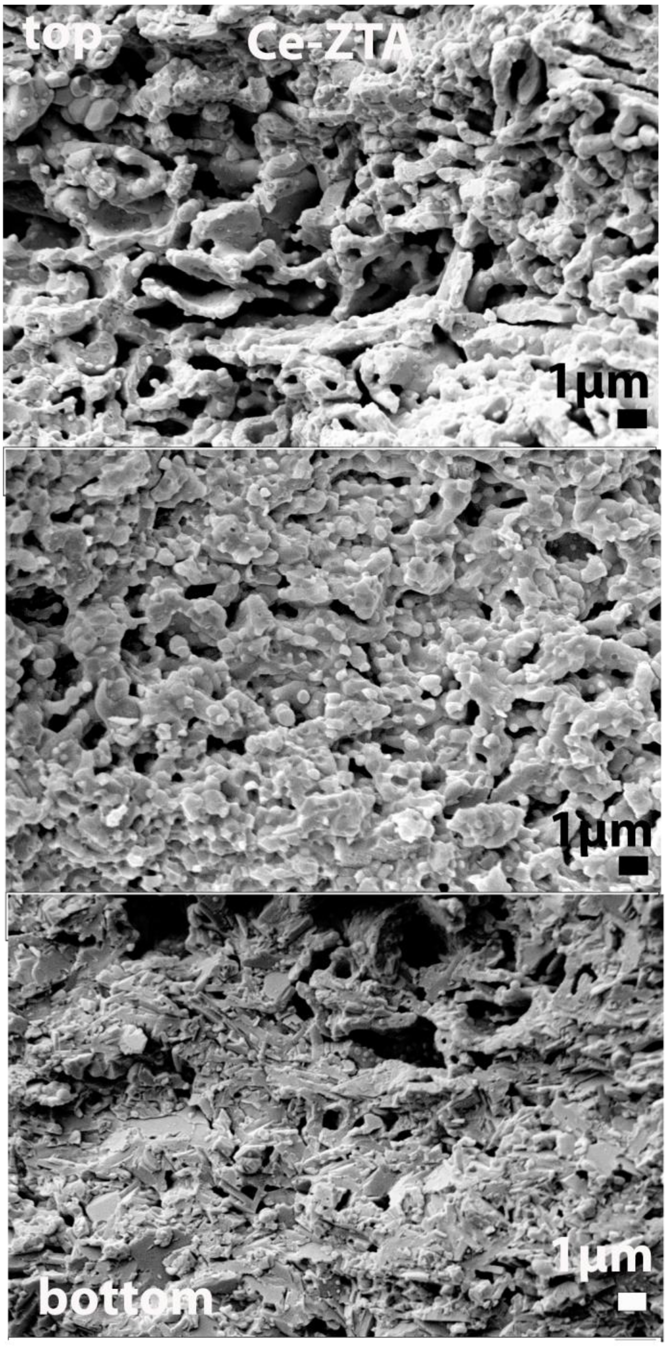

Figure. S3. SEM micrographs of the fractured surface of the cross-section of Ce-ZTA samples fabricated in ASY graphite arrangement at 1300 ºC sintering temperature
